# Role of T-Cell Polarization and Inflammation and Their Modulation by n-3 Fatty Acids in Gestational Diabetes and Macrosomia

**DOI:** 10.1155/2016/3124960

**Published:** 2016-05-24

**Authors:** A. Hichami, O. Grissa, I. Mrizak, C. Benammar, N. A. Khan

**Affiliations:** ^1^INSERM U866, Université de Bourgogne, 21000 Dijon, France; ^2^Service de Physiologie et Explorations Fonctionnelles, Faculté de Médecine de Sousse, 4000 Sousse, Tunisia; ^3^Laboratoire des Produits Naturels (LAPRONA), Département de Biologie Moléculaire et Cellulaire, Faculté des Sciences, Université Abou Bekr Belkaid, 25000 Tlemcen, Algeria

## Abstract

Th (T helper) cells are differentiated into either Th1 or Th2 phenotype. It is generally considered that Th1 phenotype is proinflammatory, whereas Th2 phenotype exerts anti-inflammatory or protective effects. Gestational diabetes mellitus (GDM) has been associated with a decreased Th1 phenotype, whereas macrosomia is marked with high expression of Th1 cytokines. Besides, these two pathological situations are marked with high concentrations of inflammatory mediators like tumor necrosis factor-*α* (TNF-*α*) and interleukin-6 (IL-6), known to play a pivotal role in insulin resistance. Dietary n-3 polyunsaturated fatty acids (n-3 PUFAs) may exert a beneficial effect by shifting Th1/Th2 balance to a Th2 phenotype and increasing insulin sensitivity. In this paper, we shed light on the role of T-cell malfunction that leads to an inflammatory and pathophysiological state, related to insulin resistance in GDM and macrosomia. We will also discuss the nutritional management of these pathologies by dietary n-3 polyunsaturated fatty acids (PUFAs).

## 1. Introduction

Maternal diabetes during pregnancy, also called gestational diabetes mellitus (GDM), is an important risk factor for foetal overgrowth, termed macrosomia, which is influenced by maternal hyperglycemia and endocrine status through placental circulation [1].

In humans, macrosomia has generally been defined as a birth weight greater than or equal to the 90th percentile birth weight for gestational age, that is, infants who weigh >4000 g at delivery, regardless of gestational age or sex [2–4]. Infants born to diabetic mothers are at an increased risk for hypoglycaemia, respiratory distress syndrome, hyperbilirubinemia, and hypertrophic cardiomyopathy [3]. It seems that maternal hyperglycaemia leads to foetal hyperglycaemia, which stimulates foetal pancreatic islet cells and, consequently, induces foetal hyperinsulinaemia. Moreover, there exists a correlationship between maternal and foetal plasma cholesterol levels in 5-6-month-old human foetuses [5, 6]. It is noteworthy that several alterations in the metabolism of carbohydrates and lipids, observed in newborn babies of diabetic mothers, also persist postnatally [7–9].

## 2. *In Utero* Programming Is Responsible for Alterations Observed in Adulthood

It is possible that foetal hyperinsulinaemia may be an endogenous teratogen factor during critical periods of foetal development, leading to permanent structural or functional changes and consequent programming of “metabolic memory.” Hyperinsulinemia* in utero* may affect the induction and activity of various hepatic enzymes associated with fat and carbohydrate metabolism [10]. This phenomenon may be accompanied, in placenta, by modifications in the expression of the transcriptional factors such as sterol regulatory element binding protein-1c (SREBP-1c), known to induce expression of genes involved in lipogenesis [11].

In 1995, Barker [12] proposed that disproportionate foetal growth induced by foetal malnutrition, which could happen either in the middle or in a later period of the gestation, programmed coronary diseases in adulthood. The* in utero* programming seems to create a kind of “metabolic memory” as the physiological abnormalities, observed during the gestational period, are responsible for the induction of diseases associated with the metabolic syndrome such as type 2 diabetes (T2D) and obesity in adulthood. Indeed, in human GDM, there exists a correlationship between GDM and 6-month-old foetal plasma cholesterol levels and other abnormal lipid parameters, including high concentrations of triglycerides (TAG), apoB100, very-low-density lipoprotein (VLDL), and low-density lipoprotein (LDL), which often persist in macrosomia [2].

Leptin, an adipocyte-derived hormone, by decreasing food intake and increasing energy expenditure, stabilizes body adiposity [13]. A transient increase in leptin during neonatal life, called “neonatal leptin surge,” has been shown to exert a neurotrophic effect and the development of energy-regulation circuits in mouse hypothalamus [14]. Furthermore, experimental premature leptin surge “from day 5.5 to day 10.5 of life” in mice pups led to decreased hypothalamic leptin sensitivity and accelerated weight gain when pups were fed a high-fat diet [15]. In healthy women, both maternal and foetal leptin concentrations were correlated with infant size at birth. Reference [16] reported that leptin levels were always higher in overweight than in normal weight newborns, and plasma leptin level was correlated with birth weight. Hence, it is conceivable that foetal leptin plays a role in* in utero* programming.

*In utero *nutritional environment may induce epigenetic alterations in the foetus. Epigenetic regulation is mediated by methylation and acetylation of histones. As far as foetal nutrition is concerned, it has been reported that dietary methyl-group intake (choline, methionine, and folate) during critical periods of development can alter DNA and histone methylation which may result in lifelong changes in gene expression [17]. Hence, these epigenetic mechanisms might contribute to the development of macrosomia and its related adulthood pathologies such as obesity and T2D [18].

Growth factors might be implicated in GDM and in the pathology of macrosomia* via* materno-foeto-placental axis. We have conducted a clinical study in which we have determined circulating growth factors and the expressions of their genes in placenta in GDM mothers and their macrosomic babies. We observed that serum concentrations of IGF-I, IGF-BP3, EGF, FGF-2, and PDGF-B were higher in GDM dames and their macrosomic babies as compared to their respective controls. Besides, the placental expression of the mRNA of growth factors (FGF-2 or PDGF-B) and growth factor receptors, that is, IGF-IR, EGFR, and PDGFR-beta, was upregulated in GDM women compared to controls [19].

## 3. Cell-Mediated Immunity in Diabetic Pregnancy and Macrosomia

The immune system is composed of two major subdivisions, the innate (phagocytic cells and NK cells) and adaptive immune responses. Though the interactions between innate and adaptive immunity are complex, the innate mechanisms control both the initiation and the type of adaptive response (Th1/Th2). It has been shown that the abnormalities in humoral and cell-mediated immunity in T1D females may persist during pregnancy and, hence, may complicate immune-foetal interaction [20].

About 10% of all GDM women develop T2D after delivery. Though pancreatic autoimmunity does not seem to represent a typical marker of GDM [21], high prevalence of autoantibodies, such as anti-GAD65 and anti-IA2 antibodies, has been observed in GDM subjects [22]. Maternal transmission of these autoantibodies did not affect diabetic risk in the offspring. On the contrary, it has been reported that foetal exposure to maternal T2D protects offspring, during the first 2 decades of life, from the development of islet autoimmunity and diabetes in the later life [23]. This is explained by the fact that the immune systems of children receiving autoantibodies from their diabetic mothers are primed during foetal life [24]. Such priming is demonstrated by an increase in MHC class II positive lymphocytes in infants of diabetic mothers compared to controls [25].

With regard to T-cell activation, only a few studies are available on the subject [20]. In humans, fully activated T-cells are detected in the cord blood of infants and mothers with T1D, but not in infants from normal mothers [20]. Interleukin-2 (IL-2) is a potent T-cell mitogen. Badr et al. [26] have reported a decrease in T-cell proliferation, associated with a decrease of plasma levels of IL-2 in the offspring of diabetic mothers, as compared to those of control mothers. Also, in rat model, probably because of their priming,* ex vivo* T-cell proliferation is significantly lower in diabetic pregnant rats and their macrosomic offspring, as compared to control animals [27]. This phenomenon may trigger a decrease in the number of circulating and thymus homing T-cells [26].

In addition, the number of T- and B-cells in the neonates of diabetic mothers was significantly decreased compared to the neonates of healthy mothers [28]. Lapolla et al. [29] reported an increase in the number of lymphocytes but a decrease in natural killer (NK) subset in children from GDM mothers. Another alteration in lymphocyte subset pattern is observed in GDM mothers, who had high number of CD8^+^ cells, expressing TCR gamma/delta, and low number of CD3^+^ cells, expressing TCR alpha/beta. Also, infants born to GDM women had higher CD8^+^ gamma/delta cells than control babies [21]. This immunological imbalance may correlate with a greater risk for developing T1D, later in life [21].

**Figure 1:**
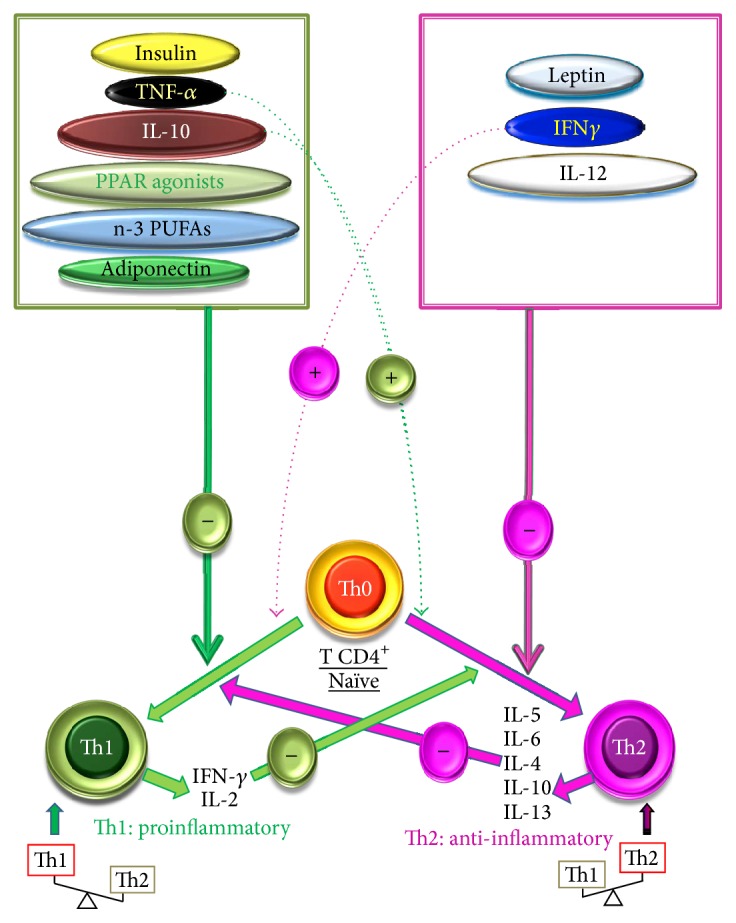
Differentiation of Th0 cells into Th1 and Th2 cells and their modulation. The secretion of their respective cytokines identifies the cells. Th0 cells, which principally secrete IL-2 along with other some cytokines, are differentiated either into Th1, under the action of IL-12 and IFN-*γ*, released by the macrophages and NK cells (natural killer), respectively, or into Th2 phenotype by the action of IL-4 produced by the mastocytes. The IFN-*γ* and IL-10 exert an inhibitory effect on the differentiation of Th1 and Th2 phenotypes, respectively. Insulin, PPAR agonists, and n-3 PUFAs promote the differentiation into Th2 phenotype. Leptin promotes the differentiation into Th1 cells. (+) inducing effect; (−) inhibitory effect.

T helper (Th) dichotomy in GDM and macrosomia has not yet been well explored. On the basis of production of cytokines, Th cells can be classified into two principal populations, Th1 and Th2 (Figure 1). Th1 cells support cell-mediated immunity and, as a consequence, promote inflammation, cytotoxicity, and delayed-type hypersensitivity, whereas Th2 cells support humoral immunity and downregulate the inflammatory actions of Th1 cells [30]. Th1 cells secrete IL-2, IFN-*α*, and TNF-*β* while Th2 cells secrete IL-4, IL-5, IL-6, IL-10, and IL-13 [31]. Analysis of T-cell markers in placenta showed an increase in T-cell infiltration that expresses GATA3, a marker of Th2 phenotype, in placenta of GDM women [32].

Concerning experimental models of GDM, we would like to mention that we have developed a model by administrating streptozotocin (STZ) to Wistar female rats [2, 9]. The rate of success in obtaining macrosomic pups was 75%. We confirmed in this model a decrease in Th1 cells, as observed in human GDM [33]. Furthermore, in GDM rat, the decrease in circulating IFN-*γ* was accompanied with an increase in IL-10 (Th2 marker) levels, as compared to control rats [34, 35]. This upregulation of Th2 phenotype in pregnancy is normalized after the delivery [34]. In fact, the shift from the Th1 phenotype to the Th2, during pregnancy, has been shown to encourage vigorous production of antibodies which not only combat infections during pregnancy, but also offer passive immunity to foetus [36]. A low Th1 profile in diabetic pregnant rats, associated with successful pregnancy, may also result from the elevated levels of reproductive hormones like human chorionic gonadotrophin (hCG) hormone, whose administration is known to diminish the production of the Th1 cytokines [37].

In rats, the upregulated Th1 profile in macrosomic animals may be due to difference in physiological status between GDM dames and their offspring [38]. A study conducted on Tunisian women with GDM and their macrosomic babies corroborates these experimental observations [39]. Indeed, the comparison of Th1/Th2 ratio showed an increase in the Th2 phenotype in GDM mothers, whereas an increase in Th1 phenotype was observed in macrosomic infants [39].

The regulatory T (T-reg) cells represent a specialized population of T-cells (CD4^+^CD25^+^), known for their properties as potent suppressors of inflammatory responses and for their ability to mediate immune tolerance. T-reg cells induce immune tolerance throughout the production of two immunosuppressive cytokines: TGF-*β* and IL-10 [40]. Both in humans [41] and in mice [42], T-regs cells increase very early in pregnancy, a period which coincides with an intense vascular activity [43]. The importance of T-reg cells in the success of pregnancy was demonstrated by Aluvihare et al. [44] who reported that adoptive transfer of T-lymphocytes depleted of T-regs cells into pregnant T-cell-deficient mice led to the rejection of allogeneic foetal units. Furthermore, spontaneous abortion cases and patients with recurrent miscarriage are associated with lower systemic T-reg cells compared to normal pregnancies [45]. In Kuwaiti women, high number of T-cells expressing the activation-associated HLA antigen (CD4^+^HLA-DR), memory T-cells, and T-reg cells have been observed during GDM [46, 47].

The frequency of T-reg cells is significantly higher in children born to T1D mothers than in those born to GDM or normal women [48, 49]. Indeed, in the case of T1D, the maternal autoimmunity and the transplacental passage of auto-GAD antibodies may influence the generation and expansion of foetal T-reg cells, which may suppress the GAD65-specific T-cell responses [48]. Besides, the T-reg cells of children born to T1D mothers exhibit a more pronounced memory phenotype (increased CCR4 expression and downregulation of CD62L), suggesting an early activation of the foetal immune system, as a consequence of maternal autoimmunity [48]. It seems that the suppressive activity of T-reg cells was significantly reduced in GDM patients when compared to healthy pregnancy [50].

It is noteworthy that obesity-induced insulin resistance is associated with the development of a specialized T-reg population in visceral adipose tissue, called “VAT resident T-reg” [51]. Visceral adipose inflammation and insulin resistance have been associated with a dramatic reduction in VAT T-reg cells in several animal models of obesity. T-reg cells, by secreting IL-10, decrease the inflammatory state of adipose tissue and, thereby, improve insulin resistance [52]. Loss-of-function and gain-of-function experiments demonstrated that VAT T-reg cells are indispensable to reducing inflammation and increasing insulin sensitivity [52]. Hence, the implication of VAT resident T-reg cells deserves deep investigations in macrosomia.

## 4. T-Cells Present a Defect in Calcium Signaling in Diabetic Pregnancy and Macrosomia

During T-cell activation, an increase in intracellular free calcium concentrations, [Ca^2+^]i, is one of the earliest events which is triggered as a result of the hydrolysis of phosphatidylinositol-bisphosphate, catalyzed by the phospholipase C (PLC). Hence, PLC gives rise to inositol trisphosphate, which recruits calcium from endoplasmic reticulum pool, and diacylglycerol which activates the protein kinase C. According to capacitive model of calcium entry, first calcium is released* via* T-cell receptor (TCR) activation from the endoplasmic reticulum (ER) and then it is extruded into the extracellular medium. In turn, the cells refill their intracellular emptied pool by opening calcium channels [49]. Ionomycin opens calcium channels, leading to calcium influx from extracellular medium and thapsigargin (TG) recruits calcium which belongs to endoplasmic reticulum (ER) pool. Interestingly, ionomycin-induced increases in [Ca^2+^]i in T-cells of GDM dames and their macrosomic offspring were greater than those in control rats [27]. In 0% of calcium buffer, TG induces increases in [Ca^2+^]i exclusively from ER pool and no influx occurs in the absence of calcium from the extracellular medium [53]. Hence, both in 100% and in 0% calcium media, TG-induced increases in [Ca^2+^]i in T-cells are higher in GDM dames and macrosomic rats than those in control animals [27], demonstrating that T-cell calcium signaling is altered in these two pathological situations.

## 5. Proinflammatory Adipokines and Cytokines in GDM and Macrosomia

TNF-*α* and IL-6 represent the main inflammatory cytokines increased in the insulin-resistant states of obesity and T2D [26, 54]. Increasing evidence suggests that GDM is a proinflammatory state similarly to T2D. Monocyte chemotactic protein-1 (MCP-1) is known to be elevated in inflammatory diseases like arthritis and lupus [55]. The elevation of MCP-1 in the third trimester of GDM suggests an association between inflammation and GDM [56]. Besides, it has been suggested that hyperglycaemia and its related oxidative stress are usually associated with increased proinflammatory cytokines production [57, 58].

**Figure 2:**
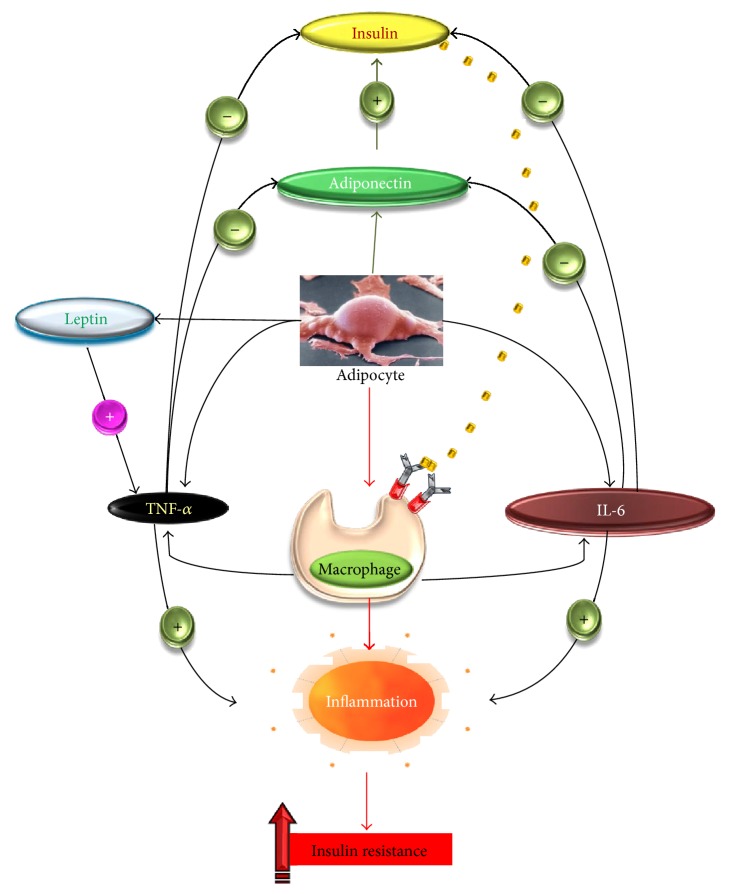
Secretion of cytokines and adipokines and their implications in insulin resistance. The adipocytes secrete adipokines (leptin and adiponectin). Proinflammatory cytokines released by macrophages. Leptin contributes to inflammation by increasing the secretion of TNF-*α*. Both TNF-*α* and IL-6 antagonize the action of insulin and decrease the secretion of adiponectin which exerts insulin-sensitizing action. (+) inducing effect; (−) inhibitory effect.

Increased concentrations of TNF-*α* and IL-6 might not only diminish insulin sensitivity by suppressing insulin signal transduction, but also interfere with the anti-inflammatory effect of insulin (Figure 2) [54, 59]. Indeed, insulin exerts its anti-inflammatory effect by decreasing the production of reactive oxygen species (ROS) from mononuclear cells and nuclear NF-*κ*B translocation [54]. Furthermore, insulin decreases the concentration of MCP-1, PAI-1, and EGR-1 [60]. The* in vivo* administration of insulin not only decreases the severity of T2D, but also diminishes the levels of MCP-1 and C-reactive protein (CRP), the two indicators of the inflammatory state [61]. IL-6 promotes insulin resistance in liver cells [62] and negatively regulates insulin signaling and glucose metabolism in adipocytes [63]. TNF-*α* inhibits tyrosine phosphorylation of insulin receptor and, thereby, insulin signaling [64]. It has been suggested that the increase in TNF-*α* and IL-6 in diabetic conditions might be a result of oxidative stress and inflammatory changes caused by hyperglycaemia [65]. IL-6 and TNF-*α* are mainly produced by adipose tissues. Indeed, during insulin-resistant state, adipocytes secrete MCP-1 which favors the infiltration of macrophages that, consequently, produce IL-6 and TNF-*α* in high quantities (Figure 2) [66, 67]. We have reported that TNF-*α* and IL-6 are increased in GDM women [39]. During pregnancy, IL-6 secretion has been proposed to aggravate insulin resistance and participates in the pathogenesis of GDM [59].

Adipocytes secrete a number of molecules, including adiponectin, leptin, and resistin, that modulate peripheral insulin sensitivity [66]. From the immunological point of view, adiponectin exhibits anti-inflammatory properties [66] and leptin polarizes Th cytokine production toward a proinflammatory (Th1) phenotype (Figure 1) [68]. Since adipocytokines may play an important role in the early defects of T2D [69], women with GDM represent an ideal population model for studying this interrelationship [70]. Leptin is also produced by placenta and involved in weight regulation and lipid metabolism. Contradictory results have been reported on its secretion in GDM. Depending on studies, elevated [71], constant [72], or decreased [73] levels of leptin have been observed in GDM women. Hence, in contrast to obesity which leads to an inflammatory Th1 state, leptin does not play an important role in GDM, marked with Th2 response, probably because of the hormonal status during pregnancy. Besides hyperglycaemia, chronic foetal hypoxia, detected in GDM, may also increase the inflammatory burden incurred by the foetus [74].

## 6. n-3 PUFAs Exert Beneficial Effects in Macrosomia

Dietary n-3 PUFAs have been considered as immunosuppressors and, therefore, are used in the management of a number of inflammatory and autoimmune diseases, including rheumatoid arthritis and multiple sclerosis [75, 76], as these pathologies are characterized by the presence of activated T-cells and inflammatory cytokines either at the site of tissue injury [77, 78] or in blood circulation [79, 80]. Fat-1 transgenic mice, known to convert endogenous n-6 PUFAs to n-3 PUFAs, were protected from diabetes, because of low concentrations of TNF-*α* and IL-1*β* [81]. Generally, both in animal models and in humans, n-3 PUFAs decrease TNF-*α* and IL-6 production [82]. n-3 PUFAs exert their effect on the inflammatory gene expression through the inhibition of intracellular signaling pathways that lead to NF-*κ*B activation [83].

n-3 PUFAs have been shown to suppress mitogen-stimulated proliferation of lymphocytes isolated from lymph nodes [84], spleen [85], and lymphatic duct [86], in mice and human beings [75, 85]. Feeding the n-3 PUFA-diet corrected intracellular calcium homeostasis in T-cells of diabetic pregnant dames and their macrosomic obese rats [27]. We have assessed the Th1/Th2 dichotomy by dietary n-3 PUFAs in diabetic pregnancy and macrosomia. We observed that the n-3 PUFAs-diet upregulated the Th2 profile in GDM rats. In macrosomic offspring, the Th1 phenotype is upregulated and an n-3 PUFAs-diet downregulated this phenomenon [33]. In agreement with our finding, Wallace et al. [76] have also observed that feeding fish oil to mice induced a shift in the IFN-*α*/IL-4 ratio, by a factor of four, as compared to animals fed the low fat diets. n-3 PUFAs also regulated T-reg functioning. We have investigated the molecular mechanisms by which n-3 PUFAs-diet controls T-reg cell suppressive capacity. We used docosahexaenoic acid (DHA), the end product of *α*-linolenic acid metabolism in animal tissues, and observed that the exposure of T-reg cells to this fatty acid or its* in vivo* supplementation upregulated TGF-*β* but downregulated IL-10 in these cells, suggesting that DHA might be orienting the T-reg cell differentiation toward a Th3 phenotype [87]. Th3 phenotypes that infiltrate decidua are known to prevent abortion and contribute to the success of pregnancy [34].

Furthermore, DHA diminished the suppressive activity of T-reg cells on effector T-cell proliferation. It is now becoming clear that the interaction of Foxp3 with other transcription factors (like NAFT or Runx-1) or histone deacetyltransferase and class II histone deacetylase is critical for the repression of the transcription of IL-2 gene by Foxp3 [88]. Hence, we hypothesized that DHA might downregulate T-reg cell activity, by interfering with the critical downstream components of the Foxp3-driven suppressor pathway. Furthermore, DHA reduced the migration of T-reg cells toward chemokines by downregulating the expression of chemokines receptors (CCR-4 and CXCR-4) in these cells [87].

T-reg cell migration and activity have been found to be associated with mitogen-activated protein kinase (MAPK), that is, ERK1/2, activation [89]. Besides ERK1/2, the phosphatidylinositol-3-kinase (PI3K) and Akt/protein kinase B (hence referred to as Akt) play a critical role in the T-cell survival, expansion, and differentiation [90]. ERK1/2 and Akt phosphorylation controls the expression of p27^KIP1^, an inhibitor of cyclinE/cyclinD kinase 2 that regulates cell cycle [91]. We noticed that DHA significantly diminished the MAPK phosphorylation in activated T-reg cells, and this phenomenon was associated with an increase of p27^KIP1^ in T-reg cells. Hence, DHA seems to reinforce the anergic state of T-reg cells [87].

Concerning the molecular mechanism of action of n-3 PUFAs, we have previously shown that dietary n-3 PUFAs are incorporated into plasma membrane phospholipids [92]. Hence, we assume that dietary n-3 PUFAs may exert their beneficial action by modulating cell signaling. We have recently shown that T-cell activation and T-cell calcium signaling are altered in diabetic pregnancy and macrosomia, and dietary fish oils, particularly eicosapentaenoic acid (EPA) and DHA, restore these T-cell abnormalities [93]. During cell activation, a modification in the intracellular pH also plays an important role in the cell cycle progression and, hence, DHA and EPA have been shown to modulate this phenomenon. Dietary n-3 PUFAs, incorporated into plasma membrane, may also give rise to diacylglycerols which, in turn, may modulate cell activation. It has been shown that diacylglycerols, containing EPA and DHA, modulate PKC activation [94], calcium signaling [95], and ERK1/ERK2 phosphorylation [96].

## 7. Conclusion

The incidence of GDM and macrosomia continues to grow worldwide and represents a major public health challenge. Except for genetic factors, physical inactivity and high caloric food are the major causing factors for these pathologies. Based on clinical studies, the* Dietary Guidelines for Americans 2005* report [97] and several international and professional organizations [98] have made recommendations for consumption of at least two meals, containing fish, per week or from 0.250 g to 1 g of EPA and DHA daily with a 5 : 1 ratio of n-6 fatty acid/n-3 fatty acid. There is no doubt concerning the beneficial effects of n-3 PUFAs in the improvements of hypertriglyceridemia and the reduction of cardiovascular risk [99]. Thus, the use of these fatty acids in combination with genuine drugs (lipid-lowering, anti-inflammatory, etc.) represents a new therapeutic strategy in fighting against diabetes and obesity.

## Abbreviations

Th: T helper
TNF-*α*: The tumor necrosis factor-*α*
IL-6: Interleukin-6
PPAR*α*: Peroxisome proliferator-activated receptor alpha
n-3 PUFAs: n-3 polyunsaturated fatty acids
GDM: Gestational diabetes mellitus
RDS: Respiratory distress syndrome
VLDL: Very-low-density lipoprotein
LDL: Low-density lipoprotein
apoB100: Apolipoprotein B 100
DHA: Docosahexaenoic acid
EPA: Eicosapentaenoic acid
PMNs: Polymorphonuclear neutrophils
NK cells: Natural killer cells
T1D: Type I diabetic
Anti-IA2: Islet Cell Autoantigen 512 Antibodies
IDDM: Insulin-dependent diabetes mellitus
TCR alpha/beta: Human T-cell receptor
STZ: Streptozotocin
IFN-*α*: Interferon alpha
Foxp3: Forkhead box P3
CTLA-4: Cytotoxic T-Lymphocyte Antigen 4
CCR4: C-C chemokine receptor type 4
PLC: Phospholipase C
TCR: T-cell receptor
MCP-1: Monocyte chemotactic protein-1
PAI-1: Plasminogen activator inhibitor
EGR-1: Early growth response protein-1
Runx-1: Runt-related transcription factor 1
ERK1/2: Extracellular signal-regulated kinases 1/2
PI3K: Phosphatidylinositol-3-kinase.
